# Role of Oxidative Stress in Blood–Brain Barrier Disruption and Neurodegenerative Diseases

**DOI:** 10.3390/antiox13121462

**Published:** 2024-11-28

**Authors:** Sehwan Kim, Un Ju Jung, Sang Ryong Kim

**Affiliations:** 1 School of Life Science and Biotechnology, Kyungpook National University, Daegu 41566, Republic of Korea; arputa@naver.com; 2 BK21 FOUR KNU Creative BioResearch Group, Kyungpook National University, Daegu 41566, Republic of Korea; 3 Department of Food Science and Nutrition, Pukyong National University, Busan 48513, Republic of Korea; 4 Brain Science and Engineering Institute, Kyungpook National University, Daegu 41404, Republic of Korea

**Keywords:** neurodegenerative diseases, reactive oxygen species, oxidative damage, blood–brain barrier, blood-derived protein

## Abstract

Upregulation of reactive oxygen species (ROS) levels is a principal feature observed in the brains of neurodegenerative diseases such as Parkinson’s disease (PD) and Alzheimer’s disease (AD). In these diseases, oxidative stress can disrupt the blood–brain barrier (BBB). This disruption allows neurotoxic plasma components, blood cells, and pathogens to enter the brain, leading to increased ROS production, mitochondrial dysfunction, and inflammation. Collectively, these factors result in protein modification, lipid peroxidation, DNA damage, and, ultimately, neural cell damage. In this review article, we present the mechanisms by which oxidative damage leads to BBB breakdown in brain diseases. Additionally, we summarize potential therapeutic approaches aimed at reducing oxidative damage that contributes to BBB disruption in neurodegenerative diseases.

## 1. Introduction

Neurodegenerative brain diseases, including Alzheimer’s disease (AD), Parkinson’s disease (PD), and Huntington’s disease (HD), are characterized by the progressive loss of neurons and the deterioration of cognitive and motor functions [1,2,3,4,5]. Neurodegeneration involves the progressive loss of neuronal structure and function, driven by mechanisms such as protein aggregation, oxidative stress, mitochondrial dysfunction, and chronic neuroinflammation [6,7,8]. These processes, influenced by genetic and environmental factors, disrupt cellular homeostasis and connectivity, leading to diseases such as Alzheimer’s and Parkinson’s [9,10,11]. Among these mechanisms, oxidative damage has emerged as a critical factor, with growing evidence highlighting its pivotal role in the pathophysiology and progression of these disorders [12,13,14,15,16,17,18,19,20,21,22,23]. Oxidative stress occurs when there is an imbalance between the production of reactive oxygen species (ROS) and the capacity of the body’s antioxidant defenses to neutralize them [24,25]. In the context of neurodegenerative diseases, the brain’s high oxygen consumption, abundant lipid content, and relatively low antioxidant capacity make it particularly vulnerable to oxidative stress [25,26,27]. Excessive production of ROS in the brain can lead to the oxidation of proteins, lipids, and nucleic acids, resulting in cellular dysfunction and death [13,14,15,18,20,28]. Oxidative damage not only directly contributes to neurotoxicity but also exacerbates disease progression by disrupting critical cellular processes such as mitochondrial function, protein homeostasis, and synaptic transmission [12,13,14,15,17,18,21,23,29,30,31,32,33]. Furthermore, oxidative stress is known to activate various signaling pathways that promote inflammation, further amplifying neuronal injury [16,19,22,30,34,35,36]. Due to the central role of oxidative damage in the etiology of neurodegenerative diseases, understanding the mechanisms by which ROS contributes to neuronal degeneration is crucial [12,13,16,37]. This knowledge can inform the development of therapeutic strategies aimed at reducing oxidative stress and mitigating its harmful effects on the brain [12,13,16,37].

Oxidative damage is a critical factor in the pathophysiology of neurodegenerative disorders, causing direct neuronal injury and disrupting the integrity of the blood–brain barrier (BBB) [38,39,40,41,42,43]. The BBB, a highly selective barrier, protects the brain by blocking harmful substances in the bloodstream while allowing essential nutrients to pass through [44,45,46,47,48]. High-density lipoprotein (HDL) plays a significant role in protecting the BBB from oxidative stress by reducing inflammation and promoting the clearance of ROS [49,50,51]. Additionally, HDL contributes to maintaining BBB integrity by regulating endothelial cell function and reducing permeability to harmful substances [50,52]. Dysfunctional or insufficient HDL exacerbates oxidative damage and compromises BBB integrity, further facilitating the entry of neurotoxic substances and inflammatory mediators into the brain [49,50,51]. Under conditions of oxidative stress, primarily driven by ROS overproduction, the structural and functional integrity of the BBB becomes severely compromised [38,39,40,41,42,43]. This disruption allows blood-derived proteins, typically excluded from the brain parenchyma, to infiltrate the central nervous system (CNS) [53,54,55,56]. Entry of these proteins into the brain can have deleterious effects, as they can trigger neurotoxicity and promote neuroinflammation [53,54]. For instance, prothrombin, thrombin, prothrombin kringle-2 (pKr-2), and fibrinogen, when present in the brain, can interact with neuronal and glial cells, exacerbating inflammatory responses [53,54,56,57,58,59,60,61,62,63,64]. This heightened inflammation can ultimately lead to neuronal cell death, further contributing to the progression of neurodegenerative diseases [53,54,60,63,64]. The influx of these blood-derived proteins not only contributes to the progression of neurodegeneration but also establishes a vicious cycle of ongoing oxidative stress and inflammation, further compromising the BBB and accelerating neuronal damage [54]. Understanding the mechanisms by which oxidative damage disrupts the BBB and permits the entry of neurotoxic blood components into the brain is essential for developing therapeutic strategies that preserve BBB integrity and mitigate the detrimental effects of oxidative stress in neurodegenerative diseases.

In this review, we explore the mechanisms through which oxidative damage contributes to the breakdown of the BBB in brain diseases. Furthermore, we discuss potential therapeutic strategies designed to mitigate oxidative damage and prevent BBB disruption in neurodegenerative diseases, emphasizing the critical role of BBB protection in preventing further neuronal injury and slowing the progression of these brain disorders.

## 2. Features of Oxidative Stress in the Brain in Neurodegenerative Diseases

**Figure 1:**
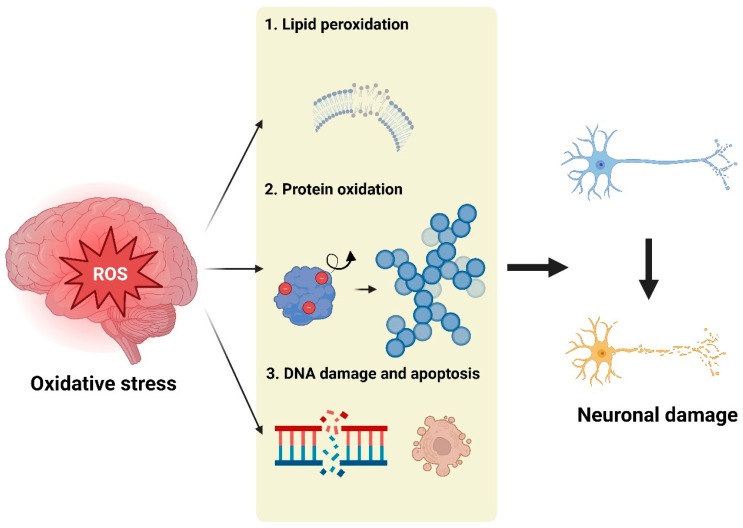
Neurotoxicity by oxidative stress in the brain. Oxidative damage in the brain leads to a cascade of detrimental effects. Lipid peroxidation destabilizes neuronal membranes, impairing their fluidity and function, which disrupts synaptic signaling and neuronal communication. Protein oxidation results in the formation and accumulation of toxic aggregates, such as amyloid plaques and tau tangles, which are hallmarks of several neurodegenerative diseases. Additionally, oxidative stress causes DNA damage, activating cell-death pathways like apoptosis. This cellular damage not only contributes to neuronal loss but also amplifies neuroinflammation, further accelerating the progression of neurodegenerative disorders. The figure was created using Biorender.com (Agreement number: NS27EOZ0V8).

Oxidative stress is a hallmark feature of the brain in neurodegenerative diseases, characterized by the excessive production of ROS that overwhelms the brain’s antioxidant defenses [13,14,16,18,20,21,28]. This imbalance leads to widespread oxidative damage, including lipid peroxidation, which destabilizes neuronal membranes, protein oxidation, which results in the accumulation of toxic aggregates, and DNA damage, which triggers cell-death pathways [35,65,66,67,68,69,70] (Figure 1). These processes contribute to the progressive loss of neuronal function and structure [35,65,66,67,68,69,70]. Additionally, oxidative stress is closely linked to the disruption of the BBB, further allowing harmful substances to penetrate the brain and exacerbate neuroinflammation, thereby accelerating disease progression [38,71,72,73].

Oxidative stress is a pivotal driver and consequence of neurodegeneration, creating a self-perpetuating cycle of cellular damage and disease progression [27,74]. It begins with an overproduction of ROS that impairs the brain’s antioxidant defenses, disrupting the balance critical for neuronal survival [26,27,75]. This imbalance results in oxidative damage to key biomolecules: lipid peroxidation destabilizes neuronal membranes, impairing synaptic signaling and producing toxic byproducts such as malondialdehyde (MDA) and 4-hydroxynonenal (4-HNE), causing further damage [76,77,78]. Protein oxidation leads to structural modifications, loss of function, and aggregation of pathological proteins, such as beta-amyloid in AD and alpha-synuclein in PD, which disrupt intracellular processes and amplify neurotoxicity [79,80,81]. ROS also induces DNA damage, particularly in mitochondrial DNA (mtDNA), which triggers cell-death pathways such as apoptosis and necrosis [82,83]. Furthermore, oxidative stress compromises the BBB, increasing its permeability to harmful substances and promoting neuroinflammation, which amplifies oxidative damage through inflammatory cytokine release and glial activation [54,84,85,86]. Neurodegeneration exacerbates these processes by impairing mitochondrial function, increasing ROS production, and weakening antioxidant defenses, thereby fueling a feedback loop of oxidative stress, inflammation, and neuronal dysfunction [6]. This multifaceted interplay underscores oxidative stress as a central mechanism in the onset and progression of neurodegenerative diseases.

### 2.1. The Generation of ROS and Its Impact

In the brain, oxidative stress primarily arises from the excessive generation of ROS, which are byproducts of normal cellular metabolism [35,67]. While mitochondria play a critical role in ATP production, electrons can leak from the electron-transport chain during oxidative phosphorylation and react with oxygen, forming ROS, which, if accumulated in excess, can lead to cellular damage [67,68,87,88]. The brain, due to its high oxygen consumption and metabolic activity, is particularly vulnerable to ROS generation [35,67].

Mitochondrial dysfunction is a common feature of neurodegenerative diseases, exacerbating ROS overproduction [67,68,87]. Under normal conditions, the ROS generated in the mitochondria are effectively neutralized by the cells’ antioxidant defense systems [67,68,87]. However, in neurodegenerative conditions, impaired mitochondrial function leads to increased ROS generation and a diminished capacity to neutralize them, resulting in the accumulation of ROS and elevated oxidative stress within brain cells [67,68,87]. ROS is highly reactive and can cause damage to essential cellular components, including lipids, proteins, and DNA [35,65,66,67,68,69,70]. Lipid peroxidation occurs when ROS attacks the polyunsaturated fatty acids (PUFAs) in cell membranes, compromising membrane permeability and structural integrity [67,68,69]. Due to the brain’s high lipid content, neuronal cells are especially susceptible to this form of damage [67,68,69]. When the cell membrane is disrupted, neurons lose their ability to function properly, eventually leading to cell death [35,65,66,67,68,69,70].

Iron plays a critical role in oxidative stress and neurodegeneration due to its unique redox properties and involvement in cellular processes [89,90,91]. While essential for brain function, including oxygen transport and mitochondrial activity, dysregulated iron homeostasis contributes significantly to oxidative damage in neurodegenerative diseases [89,92,93]. Iron acts as a catalyst in the Fenton reaction, where it reacts with hydrogen peroxide to produce highly reactive hydroxyl radicals (•OH), leading to lipid peroxidation, protein oxidation, and DNA damage [94,95,96]. These processes destabilize neuronal membranes, promote toxic protein aggregates such as beta-amyloid in AD and alpha-synuclein in PD, and trigger cellular death [80,97,98]. Furthermore, iron accumulation, which is commonly observed in neurodegenerative conditions, disrupts mitochondrial function and increases ROS production, further exacerbating oxidative stress [90,99,100]. Moreover, iron-induced oxidative damage compromises the BBB, allowing harmful substances to infiltrate the brain and intensify neuroinflammation, creating a vicious cycle of neuronal dysfunction and degeneration [85,101,102].

### 2.2. Lipid Peroxidation

Primarily due to the high lipid content of neuronal membranes, lipid peroxidation represents one of the most harmful consequences of oxidative stress within the brain [103,104,105]. The brain is particularly vulnerable to this process because of its rich concentration of PUFAs, which are essential components of neuronal cell membranes and are highly susceptible to attack by ROS [106,107]. When ROS reacts with these fatty acids, it initiates a chain reaction that produces lipid peroxides, which compromises the structural integrity and fluidity of the cell membrane [83,108]. The breakdown of cell membrane integrity is a critical factor in neuronal dysfunction, as it disrupts normal cell-signaling pathways, membrane permeability, and ion homeostasis [109,110]. This can ultimately lead to neuronal cell death, further contributing to the neurodegenerative process [109,110]. Lipid peroxidation not only damages membrane lipids but also generates toxic byproducts such as MDA and 4-HNE, which play a significant role in propagating oxidative damage [111]. These lipid peroxidation products are particularly harmful because they can form adducts with proteins and nucleic acids, leading to further impairment of cellular function [111]. For instance, MDA and 4-HNE can modify the structure of proteins through covalent bonding, disrupting protein folding and enzymatic activity [107,111]. This contributes to the accumulation of damaged and dysfunctional proteins, which is a hallmark of many neurodegenerative diseases. Moreover, these byproducts can induce DNA damage, further exacerbating cellular dysfunction and promoting neuronal cell death [111].

The cumulative effects of lipid peroxidation and its byproducts contribute to the progression of neurodegenerative diseases such as AD, PD, and amyotrophic lateral sclerosis (ALS) [112,113]. As these oxidative reactions propagate, they create a vicious cycle of membrane damage, disrupted cellular function, and neurotoxicity, all of which accelerate neuronal degeneration and exacerbate the clinical manifestations of these diseases [19,114].

### 2.3. Protein Oxidation and Aggregation

Oxidative stress leads to the oxidation of proteins, resulting in the modification of amino acid side chains, fragmentation of the protein backbone, and the formation of protein aggregates [115,116,117]. In the brain, these oxidative modifications often cause the accumulation of misfolded proteins and aggregates [98,118,119]. Prominent examples include amyloid-beta (Aβ) in AD and alpha-synuclein in PD [98,120]. These misfolded proteins accumulate and form toxic aggregates, which are highly neurotoxic [98,121]. The presence of these protein aggregates disrupts synaptic function and impairs critical cellular-signaling pathways, leading to a decline in neuronal function [122,123]. As a result, neurons become increasingly vulnerable, ultimately undergoing cell death, which significantly contributes to the progression of neurodegenerative diseases [124,125]. Moreover, these aggregates are not confined to a single cell but can spread pathologically throughout the brain, affecting surrounding neurons and exacerbating the damage [124,125]. This process is central to the pathophysiology of diseases where neuronal death and functional impairment of the brain are key features [124,125]. Protein oxidation and aggregation, driven by oxidative stress, play a crucial role in the progression of these diseases, accelerating the spread of pathology across neuronal networks and worsening clinical symptoms [98,115,117,121].

### 2.4. DNA Damage and Apoptosis

In the brain, ROS-induced oxidative stress can inflict severe damage on both nuclear and mtDNA, leading to mutations, strand breaks, and activation of DNA-repair pathways [65,66,67,82,126]. However, the brain’s inherent limitations in DNA repair capacity make it particularly susceptible to accumulating DNA damage [127,128]. In neurodegenerative diseases, persistent DNA damage overwhelms the repair mechanisms, causing genomic instability and triggering apoptotic pathways [129,130]. When oxidative DNA damage persists, it can lead to the activation of cell-death pathways such as apoptosis, which is a programmed cell-death process [83,131,132]. Apoptosis is a key mechanism in the progressive loss of neurons observed in diseases such as AD, PD, and ALS [124,131,133]. The inability to adequately repair DNA damage accelerates the degeneration of neurons, further contributing to cognitive and motor dysfunction as these diseases advance [134,135].

This continuous cycle of DNA damage, inadequate repair, and apoptosis significantly exacerbates neuronal loss, making oxidative DNA damage a critical driving force in the pathogenesis of neurodegenerative diseases [136,137,138]. As neurons are irreplaceable, their gradual loss due to ROS-induced apoptosis plays a central role in the overall progression and severity of neurodegeneration [139,140].

## 3. BBB Disruption Caused by Oxidative Damage in the Brain

The BBB is a highly selective and protective structure that separates circulating blood from the brain’s extracellular fluid, playing a crucial role in maintaining CNS homeostasis [141,142]. Composed of endothelial cells, tight junction proteins, astrocytic end-feet, and pericytes, it ensures the controlled passage of essential nutrients and specific molecules into the brain while blocking potentially harmful substances [141,142,143,144]. However, the integrity of the BBB is frequently compromised in neurodegenerative diseases, largely due to oxidative damage caused by an imbalance between ROS overproduction and the brain’s antioxidant defenses [45,46,145,146,147].

**Figure 2:**
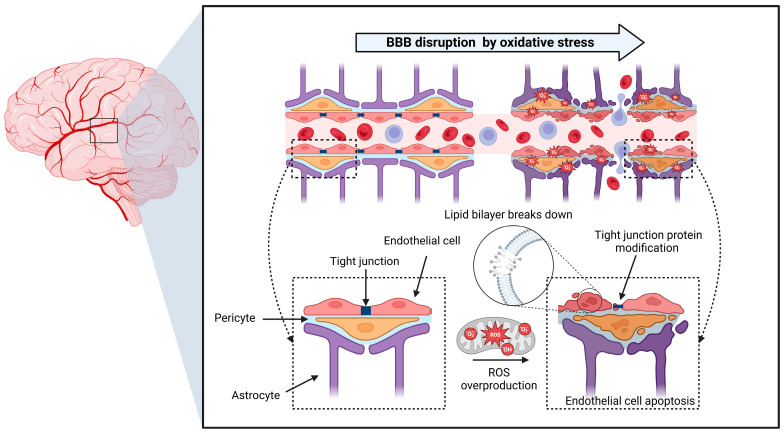
BBB disruption by oxidative stress in the brain. Oxidative stress compromises the integrity of the BBB, playing a pivotal role in neurodegenerative diseases. Excessive ROS production induces oxidative stress, which adversely affects endothelial cells and tight junction proteins essential for BBB stability and selective permeability. Lipid peroxidation disrupts cell membrane integrity, weakening barrier function, while oxidative modifications of tight junction proteins, such as claudins and occludins, impair their regulatory capabilities. Consequently, the BBB becomes more permeable, allowing neurotoxic substances, inflammatory mediators, and blood-derived proteins to infiltrate the brain parenchyma. The figure was created using Biorender.com (Agreement number: FY27LOUENC).

ROS, including superoxide anions, hydrogen peroxide, and •OH, is a highly reactive byproduct of cellular metabolism, particularly within mitochondria [67,68,87,88]. In neurodegenerative diseases, mitochondrial dysfunction leads to increased ROS production, which directly impacts endothelial cells and tight junction proteins critical for maintaining BBB integrity (Figure 2) [67,68,83,87,88,148,149,150,151]. ROS-induced lipid peroxidation destabilizes the cellular membranes of endothelial cells, while oxidative modifications to tight junction proteins such as claudin and occludin disrupt their function, increasing BBB permeability (Figure 2) [38,143,148]. This allows neurotoxic substances, including blood-derived proteins and inflammatory mediators, to infiltrate the brain, triggering neuroinflammation and exacerbating neuronal damage [54,63]. Therefore, understanding how oxidative stress compromises the BBB is essential for developing strategies to protect brain health and slow the progression of neurodegenerative diseases.

### 3.1. Lipid Peroxidation and Membrane Integrity in BBB Disruption

**Figure 3:**
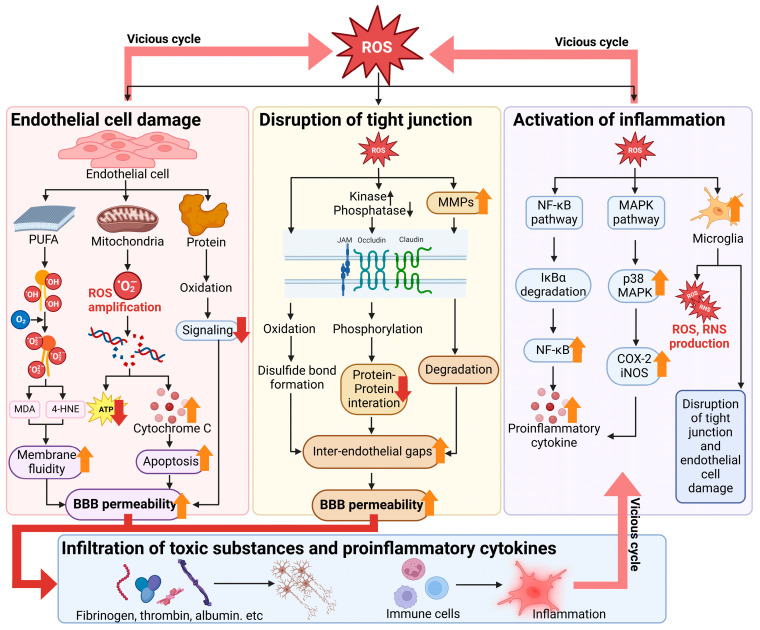
Mechanisms of BBB breakdown caused by oxidative stress in the brain. ROS plays a critical role in disrupting the BBB by targeting endothelial cells and tight junction proteins and activating inflammatory pathways. ROS attack PUFAs in endothelial cell membranes, initiating lipid peroxidation, which generates reactive aldehydes such as MDA and 4-HNE. These aldehydes covalently bind to proteins and DNA, impairing cellular functions and increasing membrane permeability. Simultaneously, mitochondrial dysfunction amplifies ROS production as leaked electrons from the electron transport chain form superoxide radicals, damaging mtDNA, reducing ATP synthesis, and triggering apoptosis via cytochrome c release. ROS further destabilizes tight junction proteins, including occludin, claudin, and ZO-1, by oxidizing cysteine residues, disrupting disulfide bonds, and inducing hyperphosphorylation through kinases such as PKC or by inhibiting phosphatases such as PP2A. This leads to structural weakening and increased permeability of the tight junction complex. Additionally, ROS activates matrix metalloproteinases (MMPs; e.g., MMP-9), which degrade tight junction proteins, widening endothelial gaps. These changes allow harmful substances, including fibrinogen, thrombin, and albumin, as well as inflammatory cytokines such as TNF-α and IL-6, to infiltrate the brain, thereby intensifying neuroinflammation. The resulting activation of pathways such as NF-κB and MAPK amplifies the release of inflammatory mediators and further ROS production, creating a vicious cycle of oxidative stress, BBB damage, and neuronal dysfunction. Addressing these mechanisms with targeted therapies, including antioxidants, anti-inflammatory agents, and metalloproteinase inhibitors, could help protect BBB integrity and prevent neurodegenerative progression. The figure was created using Biorender.com (Agreement number: LJ27LOY3MS).

One of the critical mechanisms by which ROS disrupts the BBB is through the lipid peroxidation of the endothelial cell membranes, which are rich in PUFAs [43,152]. ROS initiates lipid peroxidation, leading to the formation of lipid peroxides, which severely compromise the structural integrity of the cell membranes [83,153]. ROS, particularly •OH, targets PUFAs in the endothelial cell membrane, initiating lipid peroxidation [154,155,156]. This process generates lipid radicals, which interact with oxygen to form lipid hydroperoxides [77]. These hydroperoxides degrade into reactive aldehydes such as MDA and 4-HNE, which covalently bind to proteins and DNA, disrupting cellular functions (Figure 3) [157,158,159]. This process destabilizes the lipid bilayer, which is essential for maintaining the barrier function of endothelial cells [83,153]. As the lipid bilayer breaks down, the physical structure of the BBB is weakened, and the tight junctions between endothelial cells are compromised [83,153]. Tight junction proteins, which play a key role in regulating the selective permeability of the BBB, lose their integrity due to the breakdown of the lipid bilayer, resulting in increased BBB permeability [160,161,162]. This disruption allows potentially harmful substances, such as neurotoxic molecules and inflammatory mediators, to pass through the barrier and enter the brain [54,63]. The compromised membrane integrity from lipid peroxidation thus results in the loss of BBB function, contributing to the progression of neurodegenerative diseases by promoting neuroinflammation and accelerating neuronal damage [152,163].

### 3.2. Tight Junction Protein Modification by Oxidative Stress

Oxidative stress can induce modifications to these tight junction proteins, compromising their functions [38,162,164]. ROS can cause nitration and oxidation of tight junction proteins, leading to their dysfunction and eventual degradation [165,166]. This oxidative modification weakens the tight junctions, resulting in the loosening of the cell–cell connections between endothelial cells [38,162,164]. ROS disrupts tight junction proteins such as occludin, claudin, and ZO-1 through oxidative and post-translational modifications, including the oxidation of cysteine residues that destabilize disulfide bonds and protein interactions [167,168]. Additionally, they induce hyperphosphorylation of these proteins via the activation of kinases such as protein kinase C or inhibition of phosphatases such as PP2A, further weakening the structural integrity of the tight junction complex (Figure 3) [167,168]. ROS also promotes the expression and activation of MMPs, particularly MMP-9, which degrade occludin and claudin, widening intercellular gaps between endothelial cells (Figure 3) [160,169]. These processes collectively increase BBB permeability, enabling harmful substances to infiltrate the brain [170,171,172]. Consequently, the paracellular pathway, which is typically tightly regulated, becomes more permeable, allowing neurotoxic substances and other potentially harmful agents to pass through the BBB and enter the brain parenchyma [54,63]. This increase in permeability contributes to the initiation and exacerbation of neuroinflammation and promotes neuronal damage, playing a significant role in the progression of neurodegenerative diseases [54,63]. Understanding how oxidative stress leads to tight junction protein modification is crucial for developing therapeutic strategies aimed at preserving BBB integrity and preventing the pathological entry of harmful substances into the brain [54,63].

### 3.3. Endothelial Cell Apoptosis

Oxidative stress is a key factor that triggers apoptosis, or programmed cell death, in endothelial cells [173,174,175]. Excessive production of ROS leads to oxidative damage that overwhelms the cellular repair mechanisms, resulting in mitochondrial dysfunction and the activation of apoptotic pathways [83,151,176]. ROS generated from the mitochondrial electron transport chain (Complexes I and III) causes oxidative damage to mtDNA and proteins, such as cytochrome c (Figure 3) [82,177]. Damaged mtDNA impairs mitochondrial enzyme function, further increasing ROS production [32,178,179]. This creates a vicious cycle, reducing ATP production and causing mitochondrial permeability transition, which releases cytochrome c into the cytosol and triggers apoptosis (Figure 3) [180,181,182]. Mitochondrial damage caused by ROS not only depletes cellular energy but also creates a vicious cycle of further ROS production, exacerbating the death of endothelial cells [176,183]. As endothelial cells undergo apoptosis, the structural integrity of the BBB is severely compromised [176,183]. The loss of endothelial cells creates gaps in the BBB, through which neurotoxic substances and inflammatory mediators can infiltrate the brain [54,63]. Therefore, understanding how oxidative stress induces endothelial cell apoptosis is crucial for developing therapeutic strategies aimed at protecting the BBB and preventing neuronal damage in neurodegenerative conditions.

### 3.4. Inflammation and Oxidative Stress

The disruption of the BBB caused by oxidative damage often initiates a secondary wave of neuroinflammation, further exacerbating the progression of neurodegenerative diseases [16,152,184]. Once the BBB is compromised, blood-derived proteins, immune cells, and other inflammatory mediators infiltrate the brain, triggering an amplified inflammatory response [54]. This infiltration leads to the activation of resident immune cells, such as microglia and astrocytes, which release pro-inflammatory cytokines and additional ROS [54,59]. The sustained activation of these glial cells creates a vicious cycle where ongoing inflammation further increases ROS production, causing additional oxidative damage to neuronal and glial cells [185,186,187]. The NF–κB pathway is initiated by the degradation of IκBα, enabling NF-κB to translocate to the nucleus and promote the transcription of pro-inflammatory cytokines such as TNF-α, IL-1β, and IL-6, which further damage endothelial cells and disrupt tight junction proteins (Figure 3) [188,189,190]. Simultaneously, ROS activates MAPKs such as p38 and JNK, increasing the expression of inflammatory mediators and MMPs, which accelerate BBB breakdown (Figure 3) [191,192]. Additionally, ROS stimulates microglial activation, leading to the release of more ROS, RNS, and cytokines. This creates a feedback loop that intensifies BBB disruption and neuroinflammation (Figure 3) [152,193,194]. This cycle perpetuates BBB disruption, allowing even more neurotoxic substances to enter the brain, thus fueling inflammation and oxidative stress [152,195]. This dynamic interaction between inflammation, oxidative stress, and BBB breakdown contributes significantly to the progression of neurodegenerative diseases such as AD and PD and underscores the importance of targeting both oxidative stress and inflammation in therapeutic strategies [56,144,196].

ROS-induced endothelial and tight junction damage disrupts the BBB, allowing inflammatory mediators and additional ROS-generating substances to enter the brain [152,160,197]. This exacerbates oxidative stress and neuroinflammation [16,114]. The infiltrated substances and activated immune cells amplify ROS production, worsening BBB damage and perpetuating the pathological cascade seen in neurodegenerative diseases [172,198,199]. Thus, breaking this cycle is crucial for maintaining BBB integrity and mitigating neuroinflammation and neuronal damage [152,172,198].

## 4. Disruption of the BBB Due to Oxidative Damage Facilitates the Influx of Blood-Derived Proteins into the Brain

Once the BBB is compromised, blood-derived proteins such as prothrombin, thrombin, pKr-2, and fibrinogen can infiltrate the brain parenchyma [54,63]. The presence of these proteins in the brain is abnormal and triggers a cascade of pathological events [53,54,63,64,200]. One of the most significant consequences is the activation of microglia, the brain’s resident immune cells, which play a central role in the inflammatory response [53,54,63,64]. A study by Kim et al. (2023) specifically highlights how blood-borne proteins induce microglial activation upon entering the brain [54]. For instance, prothrombin and thrombin are involved in blood coagulation, but their increased expression in the brain, especially in AD, suggests a role in neurodegeneration [58,200]. Thrombin is known to activate microglia, promoting the release of pro-inflammatory cytokines and leading to oxidative stress, inflammation, and neuronal death [57,58]. It also contributes to Aβ accumulation and tau hyperphosphorylation, exacerbating AD pathology [54,62]. pKr-2 is a fragment released during prothrombin activation and has been shown to induce neuroinflammation through microglial activation without directly causing neuronal toxicity [54,60,61,63,64]. Moreover, pKr-2 overexpression leads to excessive neuroinflammation and neuronal death via activation of TLR4 transcription factors such as PU.1 and p-c-Jun [64]. Controlling pKr-2 overexpression has been suggested as a potential therapeutic approach to mitigate neuroinflammation and cognitive decline [54,63]. Fibrinogen plays a key role in blood clotting but has been found to cross the BBB in neurodegenerative diseases, contributing to neuroinflammation and synaptic damage [53,62]. It activates microglia, promotes the removal of synaptic spines, and is linked to vascular damage and BBB breakdown in AD [144,201,202]. Fibrin, a derivative of fibrinogen, accumulates in the brain and worsens Aβ deposition, accelerating the progression of AD [62]. Other blood-derived proteins such as albumin, immunoglobulins, and plasminogen also infiltrate the brain through a compromised BBB, triggering microglial activation and contributing to neuroinflammation and neurodegeneration [54,152,184]. MMPs, particularly MMP-9, and proteins such as HMGB1 have also been implicated in BBB breakdown, highlighting the need for further research into these pathways as potential therapeutic targets for AD [184,203,204].

The combined effects of these blood-derived proteins lead to a vicious cycle of inflammation and oxidative stress, further accelerating neurodegenerative disease progression [54,63,64]. Understanding the mechanisms by which oxidative damage disrupts the BBB and facilitates the entry of blood-derived proteins into the brain is crucial for developing therapeutic strategies to mitigate BBB disruption, neuroinflammation, and neurodegeneration.

## 5. Therapeutic Approaches for Protecting the BBB by Inhibiting Oxidative Damage

Antioxidants play a crucial role in protecting BBB integrity by counteracting the damaging effects of oxidative stress [143,196,205]. By mitigating lipid peroxidation, protein oxidation, and DNA damage, antioxidants help preserve BBB permeability and prevent the infiltration of neurotoxic blood-derived substances into the brain [150,206]. This protective effect reduces the activation of neuroinflammatory pathways and the progression of neurodegenerative diseases such as AD and PD [207,208,209]. Enhancing antioxidant defenses through dietary intake, pharmacological agents, or upregulation of endogenous antioxidant systems offers a promising strategy to protect the BBB and combat the deleterious effects of oxidative stress in the CNS [27,38,143].

Antioxidants act by neutralizing ROS, thereby preventing the oxidative modification of cellular components such as lipids, proteins, and DNA within endothelial cells. By doing so, antioxidants help maintain the structural integrity of the BBB and reduce the entry of harmful substances into the brain [143,210]. Among the most studied antioxidants are N-acetylcysteine (NAC), resveratrol, vitamin E, and alpha-lipoic acid, each of which has demonstrated significant potential in strengthening the BBB in preclinical models of neurodegenerative diseases [211,212].

NAC is a potent antioxidant that serves as a precursor to glutathione, one of the brain’s most important endogenous antioxidants [211,212]. By boosting glutathione levels, NAC enhances the brain’s ability to neutralize ROS and reduce oxidative stress [211,212]. One study has shown that NAC can inhibit lipid peroxidation and prevent oxidative degradation of tight junction proteins such as occludin and claudin, both of which are critical for maintaining BBB integrity [213]. Furthermore, NAC has been shown to attenuate microglial activation—a major driver of neuroinflammation—thereby breaking the cycle of oxidative stress and inflammatory damage [214,215]. This dual action of NAC, both in protecting the BBB and in reducing inflammation, makes it a promising candidate for neuroprotective therapy.

Another potent antioxidant, resveratrol, is a naturally occurring polyphenol found in grapes, berries, and red wine [216,217,218]. Resveratrol has garnered attention for its ability to inhibit NADPH oxidase, an enzyme responsible for generating ROS in the brain [219,220]. By inhibiting this enzyme, resveratrol reduces ROS production and prevents oxidative damage to the BBB [219,220]. Additionally, resveratrol modulates the expression of tight junction proteins, helping to preserve the selective permeability of the BBB [221,222]. In neurodegenerative models, resveratrol has also been shown to reduce the production of pro-inflammatory cytokines such as TNF-α and IL-6, thus protecting the brain from neuroinflammatory cascades that exacerbate neuronal damage [223,224,225]. Moreover, resveratrol has been linked to the promotion of autophagy, a process that helps clear damaged cells and proteins, which can further protect against oxidative damage [226].

Vitamin E, a well-known fat-soluble antioxidant, has been extensively studied for its role in protecting cell membranes from oxidative damage [227,228]. By preventing lipid peroxidation, vitamin E helps stabilize endothelial cell membranes, reducing BBB permeability [229]. In experimental models, vitamin E has been shown to reduce oxidative stress within the CNS and enhance the expression of tight junction proteins, thus restoring BBB integrity [229,230]. The ability of vitamin E to reduce the infiltration of neurotoxic proteins such as fibrinogen and thrombin into the brain has been linked to decreased neuroinflammation and improved cognitive outcomes in animal models of AD [54,229,230].

Alpha-lipoic acid is another powerful antioxidant that has been shown to protect both lipid and aqueous environments in the brain [231,232]. It works by scavenging ROS, promoting glutathione regeneration, and reducing oxidative stress in endothelial cells [231]. Studies have demonstrated that alpha-lipoic acid can preserve tight junction integrity [231] and reduce neuroinflammation by inhibiting nuclear factor-kappa B (NF-κB) signaling, a key pathway involved in the production of pro-inflammatory cytokines [233]. This antioxidant not only protects the BBB but also reduces neuronal damage by preventing the overactivation of astrocytes, which are major contributors to the chronic inflammation observed in neurodegenerative diseases [233,234].

The role of antioxidants in neuroprotection goes beyond simply neutralizing ROS [26,235,236]. By reducing oxidative stress, antioxidants help prevent the secondary wave of damage caused by glial cell activation [34,186,237]. In conditions where the BBB is compromised, blood-derived proteins infiltrate the brain and activate microglia, leading to increased ROS production and further inflammation [54,63]. This creates a vicious cycle where oxidative stress and neuroinflammation feed into each other, accelerating BBB disruption and neurodegeneration [86,114]. By breaking this cycle, antioxidants help protect not only the BBB but also neurons and synapses from further damage [238]. We also recently reported that supplying water with caffeine, which may offer potential benefits in reducing ROS in the brain, can enhance the preservation of the BBB in an animal model of AD, resulting in neuroprotective effects through anti-inflammatory responses in the CNS [53,54,60,63,64].

Previous studies have provided evidence of the neuroprotective potential of antioxidants. Preclinical research has shown that NAC and alpha-lipoic acid reduce BBB permeability and prevent oxidative damage to tight junction proteins in AD models, while resveratrol reduces pro-inflammatory cytokines and inhibits microglial activation in PD models [220,239,240]. Clinically, NAC demonstrates cognitive improvement in patients with mild cognitive impairment, and vitamin E delays functional decline in AD patients [241,242,243]. Alpha-lipoic acid offers benefits in reducing oxidative stress biomarkers and improving quality of life in conditions such as multiple sclerosis and diabetic neuropathy [244,245]. However, challenges such as limited bioavailability, poor BBB penetration, and lack of disease specificity remain. Therefore, ongoing efforts focus on developing nanotechnology-based delivery systems and combination therapies to overcome these limitations and enhance the therapeutic efficacy of antioxidants [246,247,248].

Despite their benefits, antioxidants can have side effects. NAC is generally well-tolerated but may cause gastrointestinal discomfort or nausea in some individuals. Resveratrol, while effective, has limited bioavailability, and high doses may lead to headaches or dizziness [249]. Vitamin E, in high doses, poses risks of pro-oxidant effects and bleeding disorders, and alpha-lipoic acid may occasionally cause skin irritation or mild nausea [250]. These side effects are well-documented in the literature and are supported by data from the Drugs.com database, which consolidates reliable information on drug safety and efficacy.

Combining antioxidants with complementary mechanisms of action can significantly enhance therapeutic efficacy [251,252,253]. For example, a combination of NAC and alpha-lipoic acid can boost glutathione levels and address inflammation simultaneously [212,254]. Similarly, resveratrol and vitamin E may work synergistically to reduce ROS production and stabilize endothelial membranes [255,256].

Beyond simply neutralizing ROS, antioxidants play a pivotal role in disrupting the self-perpetuating cycle of oxidative stress and neuroinflammation [16,19,27]. This cycle, characterized by BBB disruption and neuronal damage, accelerates the progression of neurodegenerative diseases [172,198,257]. Interventions such as caffeine supplementation have shown promise in complementing antioxidant therapy by preserving BBB integrity and mitigating inflammatory responses in the CNS [63,258]. A structured approach to antioxidant-based therapies focuses on efficacy, safety, and synergistic combinations, providing a comprehensive strategy to protect the BBB, reduce neuronal damage, and effectively combat neurodegenerative diseases.

To overcome the limited bioavailability of antioxidants such as resveratrol, vitamin E, and alpha-lipoic acid, various strategies have been developed to enhance absorption, stability, and delivery efficiency [232,259,260]. Nanotechnology-based systems, such as nanoparticles, liposomes, and solid lipid nanoparticles, encapsulate these antioxidants to improve their stability and facilitate efficient delivery to target tissues, which includes crossing the BBB [261,262]. Lipid-based delivery systems, including nanoemulsions and nanostructured lipid carriers, enhance solubility and gastrointestinal absorption, particularly for fat-soluble antioxidants such as vitamin E and resveratrol [263,264]. Chemical modifications, such as methylation of resveratrol or esterification of vitamin E, increase their metabolic stability and bioavailability [259,265,266]. In addition, advanced drug formulations, such as transdermal patches and mucosal sprays, provide alternative routes for systemic delivery, bypassing first-pass metabolism [267]. Co-administration with bioavailability enhancers, such as piperine, or combining antioxidants with complementary mechanisms of action, such as alpha-lipoic acid with vitamin E or resveratrol, can amplify therapeutic effects [268,269,270]. Additionally, gene- and protein-based delivery systems offer innovative approaches for targeted delivery and sustained therapeutic activity [271,272,273]. Overall, these strategies address limited bioavailability and increase the therapeutic potential of antioxidants for preventing and treating neurodegenerative diseases and conditions linked to oxidative stress.

## 6. Conclusions

**Figure 4:**
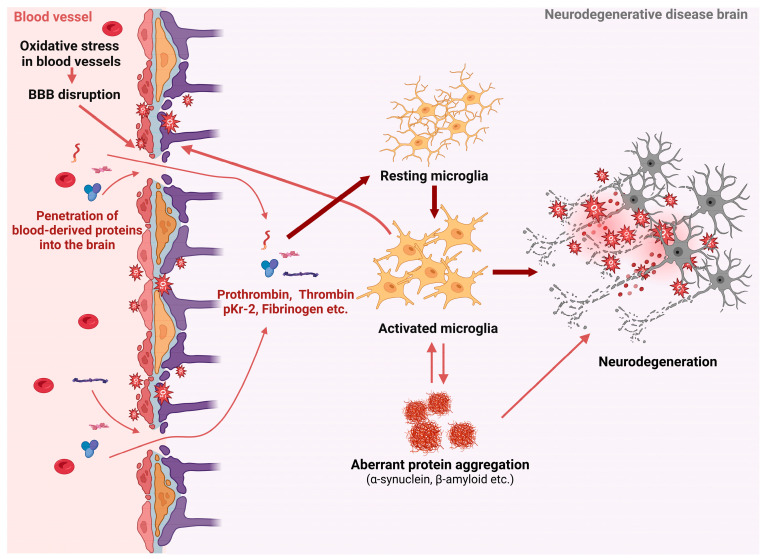
BBB disruption and neuroinflammation in neurodegenerative diseases. One of the key factors leading to the disruption of the BBB is oxidative damage, which subsequently results in increased permeability. When the BBB is compromised, blood-derived proteins such as prothrombin, thrombin, pKr-2, and fibrinogen can penetrate the brain parenchyma. A critical outcome of this process is the activation of microglia by these blood-derived proteins, which are important mediators in neuroinflammatory responses and abnormal protein accumulation. The interaction of these events consistently contributes to a harmful cycle of inflammation and oxidative stress, which intensifies and accelerates the progression of neurodegenerative diseases. The figure was created using Biorender.com (Agreement number:KA27LP17CZ).

Oxidative damage plays a pivotal role in disrupting the BBB, significantly contributing to the pathogenesis of neurodegenerative diseases by promoting neuroinflammation and neuronal injury through the influx of blood-derived proteins into the brain (Figure 4). Understanding the mechanisms by which oxidative damage undermines BBB integrity is crucial for developing targeted therapeutic strategies. Protecting the BBB from oxidative damage and preventing the entry of neurotoxic blood-derived proteins holds promise for slowing the progression of neurodegenerative diseases and improving patient outcomes. However, there are limitations such as poor bioavailability and lack of disease specificity. Emerging strategies, including nanotechnology-based delivery systems and combination therapies, aim to overcome these barriers, enhancing the efficacy and applicability of antioxidants in combating neurodegenerative diseases. Ultimately, a deeper comprehension of these processes will aid in the design of innovative interventions that could mitigate the detrimental effects of oxidative stress on the CNS, enhancing the quality of life for those affected by these debilitating conditions.
